# Comparisons between tumor burden and other prognostic factors that influence survival of patients with non‐small cell lung cancer treated with immune checkpoint inhibitors

**DOI:** 10.1111/1759-7714.13214

**Published:** 2019-11-03

**Authors:** Yoshihiko Sakata, Kodai Kawamura, Kazuya Ichikado, Naoki Shingu, Yuko Yasuda, Yoshitomo Eguchi, Jumpei Hisanaga, Tatsuya Nitawaki, Miwa Iio, Yuko Sekido, Aiko Nakano, Takuro Sakagami

**Affiliations:** ^1^ Division of Respiratory Medicine Saiseikai Kumamoto Hospital Kumamoto Japan; ^2^ Department of Respiratory Medicine Kumamoto University Hospital Kumamoto Japan

**Keywords:** Immune checkpoint inhibitor, non‐small cell lung cancer, prognostic biomarker, tumor burden

## Abstract

**Background:**

The use of baseline tumor burden (TB) as a prognostic factor for non‐small cell lung cancer (NSCLC) patients treated with immune checkpoint inhibitors (ICIs) and associations between TB and other prognostic biomarkers remain unclear. In this study, we investigated the association between TB and survival in NSCLC patients treated with ICIs in comparison with other biomarkers.

**Methods:**

We retrospectively evaluated 83 NSCLC patients with ICIs administered between February 2016 and December 2018. TB was measured as the sum of the unidimensional diameters of up to five target lesions.

**Results:**

The median observation period was 14.2 months. A total of 42 patients died during the follow‐up. Univariate Cox regression analysis showed that baseline TB was associated with OS. Cox regression analysis adjusted for Eastern Cooperative Oncology Group performance status (ECOG PS) alone or with addition of programmed cell death ligand 1 expression and treatment line showed that TB was a prognostic factor for OS. Using time‐dependent receiver operating characteristic curve analysis, the optimal TB cutoff for predicting OS was 12 cm, and patients were divided into a high TB group (*n* = 21) and a low TB group (*n* = 62). The low TB group achieved significantly longer OS than the high TB group (median OS: 18.5 months, [95% CI = 11.7‐not reached] vs. 2.3 months [95% CI = 1.3–2.9], *P* < 0.001).

**Conclusion:**

TB is a useful, clinically measurable prognostic factor of survival in NSCLC patients treated with ICIs.

## Key points

**Significant findings of the study**: Tumor burden was a prognostic factor for NSCLC patients receiving ICI treatment and was associated with overall survival not only as a categorical variable but also as a continuous variable.

**What this study adds**: Measurable biomarkers before starting ICI treatment are limited in the real‐world clinical setting. Baseline tumor burden is a clinically measurable prognostic factor for most medical institutions and can be assessed in almost any setting.

## Introduction

Immune checkpoint inhibitors (ICIs), specifically programmed cell death 1 (PD‐1)/PD‐1 ligand (PD‐L1) inhibitors, have remarkable efficacy against advanced non‐small cell lung cancer (NSCLC).^1^ Various predictive biomarkers for the response to ICIs have been previously reported, such as tumor mutation burden, mismatch repair and DNA replication genes, tumor microenvironment, immune gene signature, interferon‐γ related mRNA‐based signatures, peripheral blood biomarkers, myeloid‐derived suppressor cells, and lactate dehydrogenase (LDH) level.^2^, ^3^, ^4^, ^5^, ^6^, ^7^, ^8^, ^9^, ^10^, ^11^, ^12^ However, in the real‐world clinical setting, biomarkers that are available before starting treatment are limited. One such potential marker is tumor burden (TB). TB is calculated by adding the sum of the longest dimensions of measurable baseline target lesions, and it has been shown to be useful as a predictive biomarker for patients treated with ICIs.^13^ However, it is unclear whether TB is an appropriate prognostic biomarker for NSCLC patients treated with ICIs. In recent years, accumulating evidence has demonstrated that clinically measurable inflammatory markers are associated with a poor prognosis in lung cancer.^9^, ^14^, ^15^ However, it is unclear whether TB is superior to these other clinically measurable prognostic biomarkers. Therefore, we aimed to investigate the connection between TB and survival for NSCLC patients treated with ICIs and compare TB with clinically measurable inflammatory biomarkers.

## Methods

### Patients

We identified advanced NSCLC patients who received ICIs between February 2016 and December 2018 by searching through our hospital's prescription drug database. We enrolled all patients except for those with no measurable target lesions. Patients who were still alive at the end of February 2019 were censored; all other patients were followed‐up until death.

This study was approved by the institutional review board of our institution, and all patients provided written informed consent. Research was conducted in accordance with the 1964 Declaration of Helsinki and its subsequent amendments.

### Patient and tumor characteristics

The following variables were collected from patient electronic medical records: age, sex, Eastern Cooperative Oncology Group performance status (ECOG PS), smoking status, histology, stage, PD‐L1 expression, treatment line, type of ICI, clinically measurable inflammatory or nutritional biomarkers (such as neutrophil count, lymphocyte count, neutrophil to lymphocyte ratio [NLR], serum LDH, C‐reactive protein [CRP], albumin, and Glasgow prognostic score [GPS]), and TB. The GPS is a prognostic score that includes serum albumin and CRP levels.^16^, ^17^ NLR cutoffs were determined based on a previously published study.^9^

We measured baseline TB using computed tomography (CT) for most target lesions and magnetic resonance imaging for patients with brain metastasis, according to the Response Evaluation Criteria in Solid Tumors version 1.1. TB was defined as the sum of the longest diameters for a maximum of five target lesions and up to two lesions per organ.^13^ PD‐L1 expression assays were performed by SRL, Inc. using the Dako PD‐L1 IHC 22C3 PharmDx test.^18^

### Statistical analysis

Comparisons between groups were performed using the univariate Cox regression analysis or the Mann‐Whitney U‐test. We evaluated hazard ratios, 95% confidence intervals (CIs), and *P*‐values for each factor with an unadjusted Cox analysis. Because of the small number of deaths in this study, it was considered inappropriate to adjust for more than four factors in the multivariate Cox regression analysis used to evaluate the relative hazard of death. Accordingly, we conducted two multivariate Cox analyses. The initial analysis used variables which were significant for both the univariate Cox regression analysis and Spearman rank correlation coefficients were adjusted for tumor burden. Following this, the multivariate Cox analysis was adjusted for ECOG PS, PD‐L1 expression, and treatment line. These factors were all found to be significant risk factors for patients with NSCLC treated with ICIs in the log‐rank test and unadjusted Cox analysis in this study or other previous studies assessing ICI treatment. The most suitable cutoff level for TB was determined using time‐dependent receiver operating characteristic (ROC) curve analysis. Overall survival (OS) was defined as the time from ICI initiation to death resulting from any cause, with censoring at the last date of follow‐up. Progression free survival (PFS) was defined as the time from ICI initiation to progression of disease or death resulting from any cause, with censoring defined as the date the patient was last known to be alive and progression free. OS and PFS curves were estimated using the Kaplan‐Meier method and compared using the log‐rank test. A *P*‐value of less than 0.05 was considered statistically significant. Tumor response was evaluated based on RECIST (version 1.1) criteria.^19^

All statistical analyses were performed with EZR (Saitama Medical Center, Jichi Medical University, Saitama, Japan), a graphical user interface for R 2.13.0 (R Foundation for Statistical Computing, Vienna, Austria). More precisely, EZR is a modified version of R commander (version 1.6‐3) designed to add statistical functions frequently used in biostatistics.^20^

## Results

### Patient characteristics

We identified 88 patients who had received ICI treatment for advanced NSCLC. Five patients were excluded because they had no measurable target lesions. Hence, 83 patients who had received ICIs were included in this study. Patients were predominantly male (75%), smokers (80%), and had a good PS (ECOG PS of 0 or 1 in 84% of patients). The median observation period was 14.2 months (range 2.1–36.3 months). A total of 42 patients died during the observation period. The characteristics of the 83 patients are summarized in Table 1. There were 23 patients diagnosed with NSCLC with a PD‐L1 tumor proportion score ≥ 50% who received pembrolizumab as first‐line treatment.

**Table 1:** Patient characteristics

Characteristic	Total (%) (*n* = 83)
Age (years)	
Median (range)	69 (42–83)
Sex	
Male	62 (74.7)
Female	21 (25.3)
ECOG PS	
0	34 (41.0)
1	36 (43.4)
2	13 (15.6)
Smoking status	
Never	17 (20.5)
Current or former	66 (79.5)
Histology	
Adenocarcinoma	58 (69.9)
Squamous	18 (21.7)
Others	7 (8.4)
Driver mutation	
*EGFR* or *ALK* or *ROS1*	12 (14.5)
Others	71 (85.5)
Stage	
IV	47 (56.6)
Others	36 (43.4)
PD‐L1 expression	
≥50%	38 (45.8)
1–50%	14 (16.9)
<1%	13 (15.7)
Unknown	18 (21.7)
Treatment line	
First	23 (27.7)
Second	31 (37.3)
Third or higher	29 (34.9)

ALK, anaplastic lymphoma kinase; ECOG PS, Eastern Cooperative Oncology Group performance status; EGFR, epidermal growth factor receptor; PD‐L1, programmed cell death ligand 1; ROS1, ROS proto‐oncogene 1 receptor tyrosine kinase.

### Tumor burden is a significant predictor of overall survival

In the univariate Cox regression analysis, several baseline clinical factors were associated with survival, including ECOG PS, PD‐L1 expression, and treatment line (Table 2). For the Spearman's rank correlation coefficients, ECOG PS and histology were associated with tumor burden (Table 3). Based on these analyses the ECOG PS for the Cox regression analysis was adjusted and denoted as model 2. Additionally, the reported prognostic factors for the Cox regression analysis which included ECOG PS, PD‐L1 expression and treatment line were adjusted as model 3 (Table 4). Both model 2 and model 3 showed that TB, as a continuous variable, was a prognostic factor (hazard ratio: 1.1, 95% CI = 1.05–1.16, *P* < 0.001). Furthermore, the c‐index of TB was found to be higher than other markers used in this analysis.

**Table 2:** Univariate Cox regression analysis

Univariate Cox regression analysis	Survivors (%) (*n* = 41)	Nonsurvivors (%) (*n* = 42)	Hazard ratio	95% CI	*P*‐value
Age (years)					
Median (range)	69 (41–81)	69 (42–83)	1.008	0.97–1.05	0.71
Sex					
Male	30 (73.1)	32 (76.2)	1.05	0.52–2.15	0.89
Female	11 (26.8)	10 (23.8)	1		
ECOG PS					
0	21 (51.2)	13 (31.0)	1		
1	17 (41.5)	19 (45.2)	1.68	0.83–3.4	0.15
2	3 (7.3)	10 (23.8)	6.98	2.89–16.8	<0.001
Smoking status					
Never	9 (22.0)	8 (19.0)	1		
Current or former	32 (78.0)	34 (81.0)	1.27	0.59–2.74	0.55
Histology					
Adenocarcinoma	29 (70.7)	29 (69.0)	1		
Squamous	7 (17.1)	11 (26.2)	1.86	0.92–3.74	0.08
Others	5 (12.2)	2 (4.8)	0.5	0.12–2.13	0.35
Driver mutation					
EGFR or ALK or ROS1	7 (17.1)	5 (12.0)	0.62	0.24–1.59	0.32
Others	34 (83.0)	37 (88.1)	1		
Stage					
IV	21 (51.2)	26 (61.9)	1.19	0.64–2.22	0.59
Others	20 (48.8)	16 (38.1)	1		
PD‐L1 expression					
≥50%	27 (65.9)	11 (26.2)	1		
1–50%	7 (17.1)	7 (16.7)	3.71	1.38–9.98	0.0093
<1%	5 (12.2)	8 (19.0)	4.51	1.73–11.7	0.002
Unknown	2 (4.9)	16 (38.1)	3.96	1.83–8.54	<0.001
Treatment line					
First	18 (43.9)	5 (12.0)	1		
Second	16 (39.0)	15 (35.7)	2.58	0.94–7.11	0.067
Third or higher	7 (17.1)	22 (52.4)	4.31	1.63–11.4	0.0032
Median (range)	2 (1–6)	3 (1–6)			<0.001*

* : Comparisons between groups were performed using the Mann‐Whitney U‐test.

ALK, anaplastic lymphoma kinase; CI, confidence interval; ECOG PS, Eastern Cooperative Oncology Group performance status; EGFR, epidermal growth factor receptor; PD‐L1, programmed cell death ligand 1; ROS1, ROS proto‐oncogene 1 receptor tyrosine kinase.

**Table 3:** Correlation analysis of tumor burden and patients' background: Spearman's rank correlation coefficients

	Tumor size	
	ρ	*P*‐value
Age	−0.092	0.406
Sex: Male	0.153	0.168
ECOG PS	0.569	0.000
Smoking status: Current or former	0.214	0.052
Histology: Adenocarcinoma	−0.306	0.005
Histology: Squamous	0.344	0.001
Driver mutation: EGFR or ALK or ROS1	−0.159	0.150
Stage: IV	0.090	0.419
PD‐L1 expression*1	−0.025	0.843
PD‐L1 expression: unknown	0.038	0.730
Treatment line	−0.020	0.861

ρ: Spearman's rank correlation coefficients.

*n* = 83.

*1: Except for cases where PD‐L1 expression was unknown.

ALK, anaplastic lymphoma kinase; ECOG PS, Eastern Cooperative Oncology Group performance status; EGFR, epidermal growth factor receptor; PD‐L1, programmed cell death ligand 1; ROS1, ROS proto‐oncogene 1 receptor tyrosine kinase.

**Table 4:** Cox proportional hazard models

			Cox proportional hazard model											
				Model 1: crude			Model 2: ECOG PS adjusted				Model 3: ECOG PS, PD‐L1 and treatment line adjusted			
Marker	Survivors (%) (*n* = 41)	Nonsurvivors (%) (*n* = 42)	Reference	HR	95% CI	*P*‐value	HR	95% CI	*P*‐value	c‐index	HR	95% CI	*P*‐value	c‐index
Drug										0.744				0.761
Nivolumab	7 (17.1)	26 (61.9)	Reference	1			1				1			
Pembrolizumab	25 (61.0)	13 (31.0)		0.32	0.17–0.63	<0.001	0.22	0.11–0.46	<0.001		0.79	0.22–2.74	0.711	
Atezolizumab	9 (22.0)	3 (7.1)		0.53	0.16–1.81	0.310	0.52	0.15–1.78	0.298		0.58	0.15–2.25	0.432	
GPS										0.730				0.805
0	22 (53.7)	17 (40.5)	Reference	1			1				1			
1	9 (22.0)	8 (19.0)		1.10	0.47–2.57	0.820	1.18	0.50–2.78	0.698		2.46	0.97–6.27	0.059	
2	10 (24.4)	17 (40.5)		3.08	1.55–6.12	0.001	2.02	0.94–4.34	0.072		2.74	1.12–6.66	0.026	
Albumin (g/dL)										0.719				0.819
Median (range)	3.6 (2.3–4.6)	3.5 (1.5–4.5)	per 1 g/dL	0.39	0.23–0.66	<0.001	0.53	0.29–0.96	0.037		0.26	0.12–0.56	<0.001	
CRP (mg/dL)										0.736				0.768
Median (range)	0.64 (0.01–27.2)	1.31 (0.03–18.6)	per 1 mg/dL	1.06	1.00–1.12	0.052	1.02	0.95–1.09	0.668		1.07	0.98–1.15	0.13	
NLR										0.721				0.775
NLR <5	31 (75.6)	20 (47.6)	Reference	1			1				1			
NLR ≥5	10 (24.4)	22 (52.4)		2.88	1.56–5.29	<0.001	2.01	1.02–3.97	0.044		2.09	1.03–4.25	0.414	
Median (range)	3.15 (1.22–46.5)	5.14 (1.45–17.8)												
Neutrophil count (μL)										0.719				0.764
Median (range)	3870 (2170–30 800)	4820 (2310–19 600)	per 1000 μL	1.06	0.99–1.13	0.073	1.03	0.95–1.11	0.511		1.08	0.99–1.17	0.082	
Lymphocyte count (μL)										0.707				0.763
Median (range)	1280 (460–2700)	1110 (440–2480)	per 100 μL	0.93	0.87–1.00	0.045	0.96	0.90–1.03	0.237		0.96	0.89–1.03	0.254	
LDH (IU/L)										0.727				0.793
Normal (<223)	27 (65.9)	18 (42.9)	Reference	1			1				1			
Elevated (≥223)	14 (34.1)	24 (57.1)		2.26	1.22–4.19	0.009	2.24	1.20–4.18	0.011		2.95	1.51–5.79	0.002	
Median (range)	195 (116–360)	235 (127–2310)												
Tumor burden (cm)										0.762				0.811
Median (range)	5.4 (1–16.3)	8.8 (1.7–34.1)	per 1 cm	1.12	1.08–1.17	<0.001	1.10	1.05–1.16	<0.001		1.10	1.05–1.16	<0.001	

CI, confidence interval; CRP, C‐reactive protein; ECOG PS, Eastern Cooperative Oncology Group performance status; HR, Hazard ratio; LDH, serum lactate dehydrogenase; NLR, neutrophil to lymphocyte ratio; PD‐L1, programmed cell death ligand 1.

**Figure 1:**
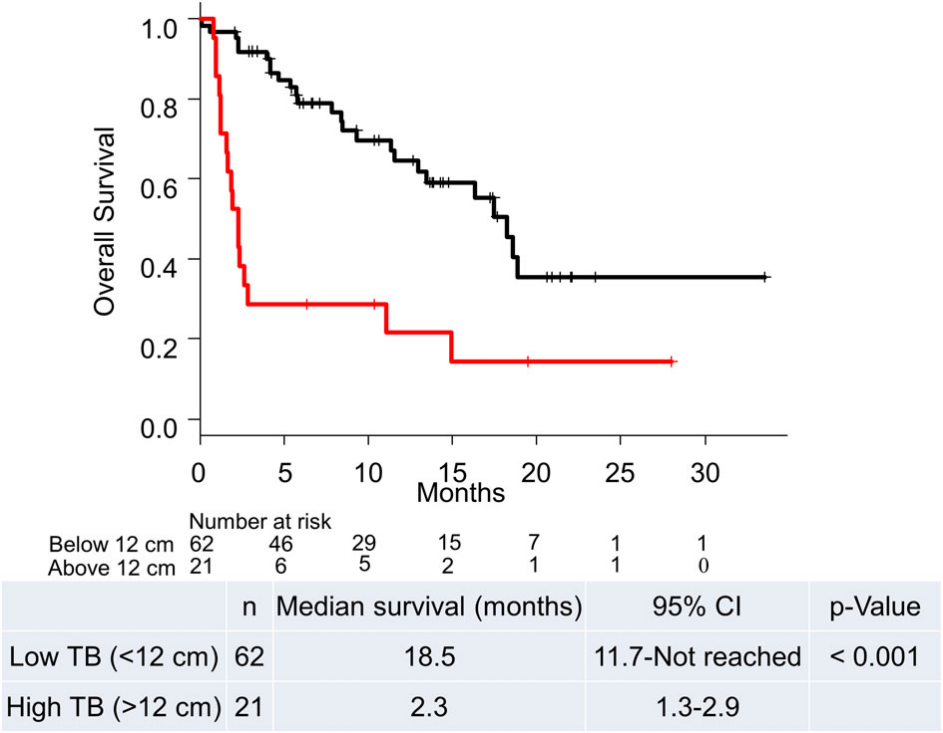
Kaplan‐Meier plot for overall survival based on tumor burden. CI, confidence interval; n, number; OS, overall survival; TB, tumor burden. (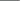) Low TB (<12 cm), and (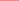) High TB (>12 cm).

**Figure 2:**
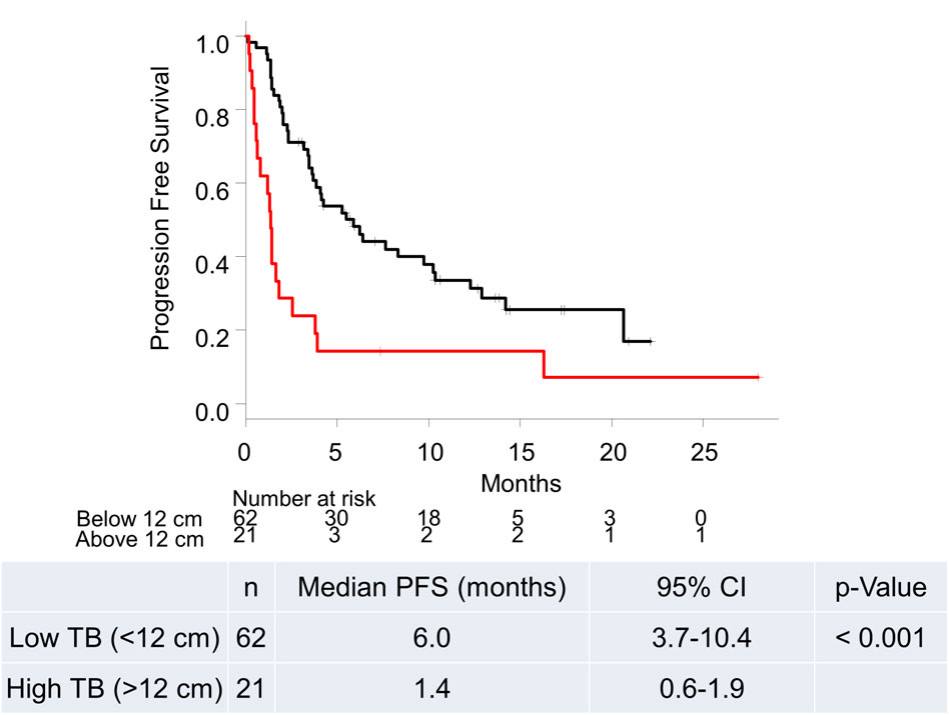
Kaplan‐Meier plots for progression free survival based on tumor burden. CI, confidence interval; n, number; PFS, progression free survival; TB, tumor burden. (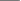) Low TB (<12 cm), and (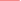) High TB (>12 cm).

Time‐dependent ROC curve analysis indicated that 12 cm was the optimal cutoff level for TB to predict survival (Figs S1 and S2). Patients were divided into a high TB group (>12 cm; *n* = 21) and a low TB group (<12 cm; *n* = 62). The median OS of patients in the high TB group was significantly shorter than that of patients in the low TB group (2.3 months [95% CI = 1.3–2.9] vs. 18.5 months [95% CI = 11.7‐not reached], *P* < 0.001; Fig 1). The median PFS of patients in the high TB group was also significantly lower than that of patients in the low TB group (1.4 months [95% CI = 0.6–1.9] vs. 6.0 months [95% CI = 3.7–10.4], *P* < 0.001, Fig 2).

## Discussion

Although many biomarkers for ICI treatment have been previously verified,^2^, ^3^, ^4^, ^5^, ^6^, ^7^, ^8^, ^9^, ^10^, ^11^, ^12^ most cannot be used in the real‐world clinical setting because they require specialized equipment or are too expensive to analyze. A recent study explored prognostic indexes based on pretreatment derived neutrophil‐to‐lymphocyte ratios and lactate dehydrogenase in patients with advanced NSCLC treated with ICIs.^21^

TB could potentially be a clinically useful prognostic factor for overall survival in NSCLC patients treated with ICI monotherapy. This study was designed to assess the utility of TB and to compare TB with other clinically measurable biomarkers. Accordingly, multivariate Cox regression analysis found that TB had a higher c‐index than other measured biomarkers. Based on this, we believe that TB reflects one of the more important factors that influences survival of NSCLC patients treated with ICIs.

There are several reasons to focus on the relationship between TB and prognosis of NSCLC treated with ICIs. First, TB is a clinically measurable biomarker for most medical institutions. Clinicians can assess TB in almost any setting as long as they evaluate the target regions by CT before starting treatment. Second, assessing TB does not require an invasive procedure, such as rebiopsy. Third, it requires no additional cost. Finally, although some previous observational studies indicated a relationship between TB and poor ICI efficacy,^13^, ^22^, ^23^ it is unclear whether TB is a prognostic factor as a continuous variable in NSCLC.

One previous study reported that pretreatment TB determined the magnitude of the drug‐induced T‐cell response: the larger the tumor, the stronger the required drug‐induced T‐cell response.^24^ Our findings suggest that both host factors and tumor factors are important. Some previous studies have already shown that host factors, such as clinically measurable inflammatory biomarkers, are associated with poor survival in lung cancer.^9^, ^14^, ^15^, ^16^, ^17^ In addition to these inflammatory biomarkers for NSCLC patients undergoing ICI treatment, we demonstrated that the tumor factor TB was a useful poor prognostic factor. As a consequence, we concluded TB was a prognostic factor associated with OS, not only as a categorical variable but also as a continuous variable.

Our results have two important implications. One is that evaluation of TB has utility in the real‐world clinical setting. The measurement method of TB in our analysis is sufficiently easy, and all patients who had measurable tumors could be assessed at any facility in which the patients could be administered anti‐cancer treatment. Therefore, the use of TB has advantages over the use of the prognostic factors that have been clarified so far. The second is that TB is a prognostic factor as a continuous variable. One previous study reported that baseline tumor size was a predictive and prognostic factor of ICI therapy in NSCLC.^23^ This study summed the longest major axis of all measurable target lesions and set the cutoff to 10.1 cm. On the one hand, our study used a more simple method that defined TB as the sum of the longest diameters for a maximum of five target lesions with a cutoff level set to 12 cm. These findings show that the appropriate cutoff levels are not defined by tumor burden. Under these circumstances, our study provided clinically useful information since we showed that higher TB volumes, when TB was considered as a continuous variable, led to more poor outcomes.

Recently, combination of chemotherapy with ICI has demonstrated superiority to chemotherapy alone.^25^, ^26^, ^27^ However, it is unclear which treatment is more appropriate, pembrolizumab alone or combination chemotherapy/ICIs for NSCLC patients with strong expression of PD‐L1 on tumor cells.^28^ Baseline TB may one of the factors that determine use of single or combination therapy.

There are some limitations to this study. First, this was a retrospective study from a single institution with a small sample size. Ideally, this outcome should be validated in a larger trial. We recommend expanding the study to multiple centers. Furthermore, there is a need to confirm whether TB is an appropriate prognostic biomarker for the combination of ICIs with cytotoxic chemotherapy. Second, there is no standard assessment for baseline TB. In this study, we calculated TB as the sum of the longest diameters of a maximum of five target lesions and up to two lesions per organ, as in a previous study.^13^ Other studies examining baseline tumor size as a prognostic factor differed with regard to measurement, and these methods were too complicated for our study.^22^, ^23^ Our main result of the association between TB and clinical outcome was comparable to those of other studies.^22^, ^23^ Therefore, we believe that it is sufficient to calculate TB as the sum of the longest diameters for a maximum of five target lesions and up to two lesions per organ. However, this method cannot be adapted for patients with no measurable target lesions. Finally, this study did not include analysis related to steroid use. Baseline steroid use in a similar study setting has been previously reported to be a negative prognostic factor.^29^, ^30^, ^31^ However, during the observation period of this study, no patient received concurrent systemic corticosteroids (equivalent to greater than 10 mg of prednisone per day) during ICI therapy as a result of our institutionally established local ICI dosing criteria.

In conclusion, we demonstrated that baseline TB was associated with survival in NSCLC patients treated with ICIs. This suggests that TB is an important clinically measurable prognostic factor, especially in patients receiving ICI treatment.

## Disclosure

The authors declare there are no conflicts of interest.

## Supporting information


**Figure S1** Time‐dependent receiver operating characteristic analysis showing an optimal cut‐off value of 12 cm for tumor burden used to predict overall survival at six months. CI, confidence interval.

**Figure S2** Time‐dependent receiver operating characteristic analysis showing an optimal cut‐off value of 12 cm for tumor burden used to predict overall survival at 12 months. CI, confidence interval.
