# Supplementary figures and images for: Comparisons between tumor burden and other prognostic factors that influence survival of patients with non‐small cell lung cancer treated with immune checkpoint inhibitors
Source: Thorac Cancer. 2019 Nov 3;10(12):2259–66. doi: 10.1111/1759-7714.13214 (PMC6885438; doi:10.1111/1759-7714.13214)

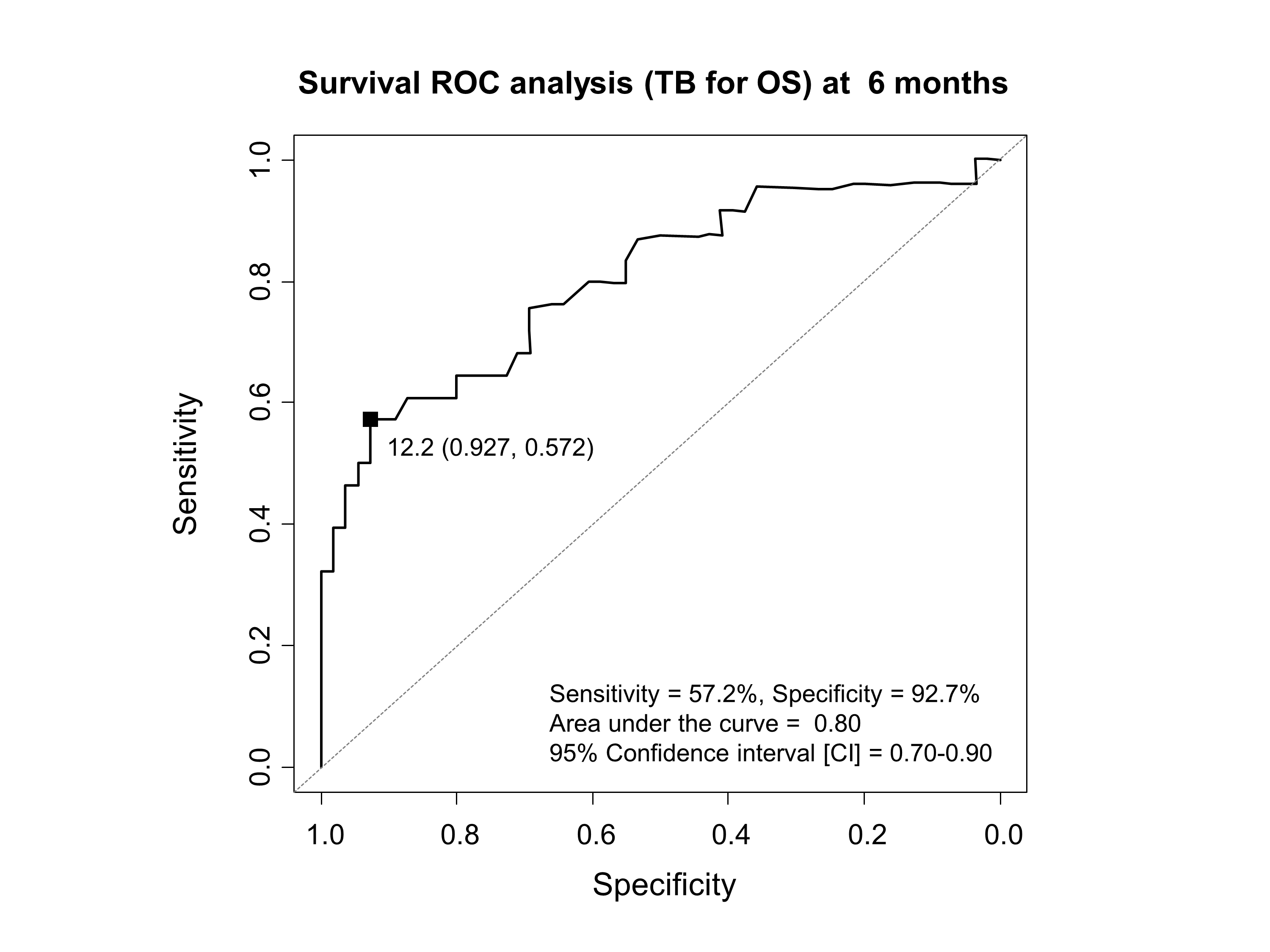

Supplement: Supplementary file 1 — Figure S1 Time‐dependent receiver operating characteristic analysis showing an optimal cut‐off value of 12 cm for tumor burden used to predict overall survival at six months. CI, confidence interval. [file TCA-10-2259-s001.tif]

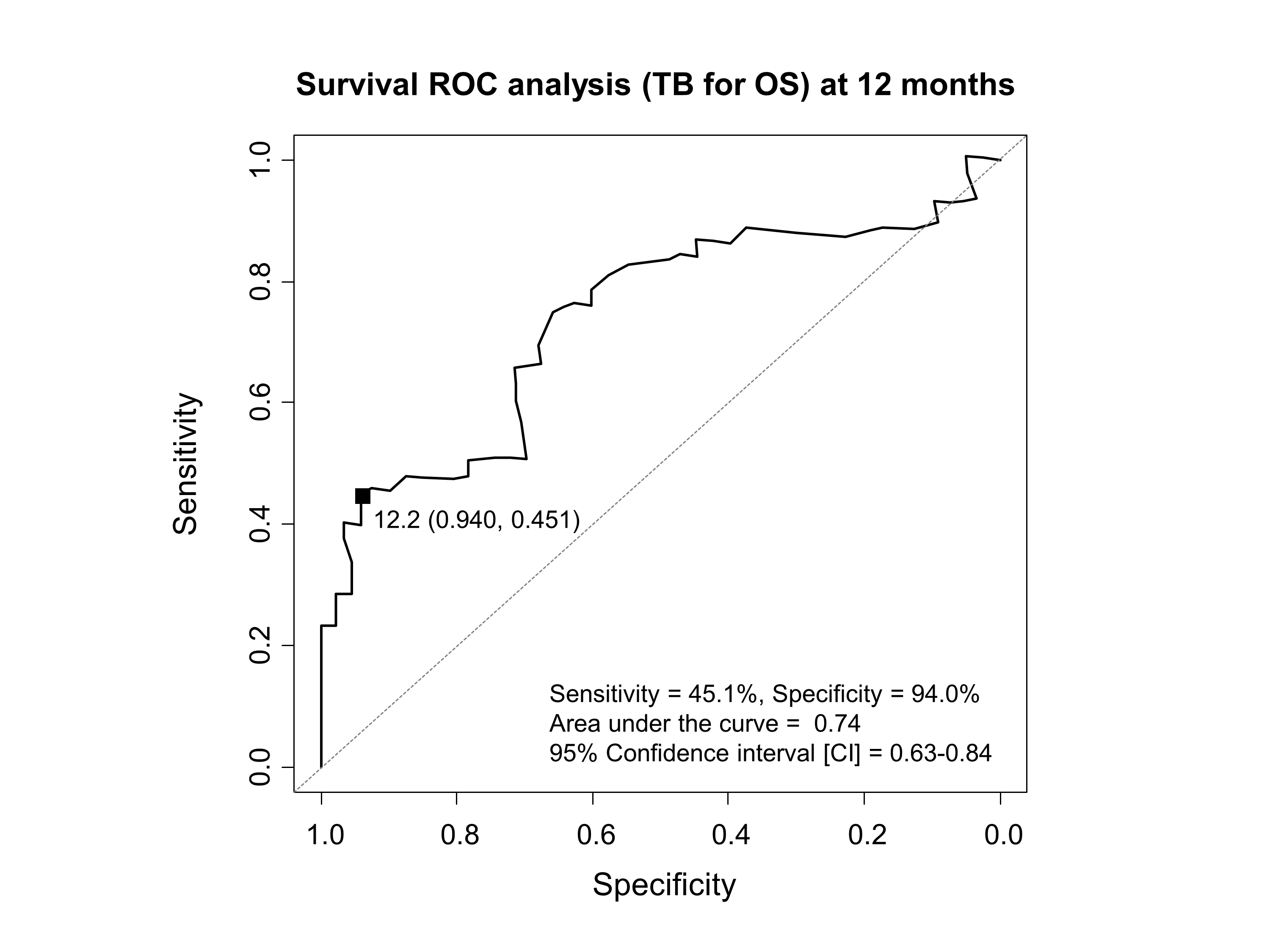

Supplement: Supplementary file 2 — Figure S2 Time‐dependent receiver operating characteristic analysis showing an optimal cut‐off value of 12 cm for tumor burden used to predict overall survival at 12 months. CI, confidence interval. [file TCA-10-2259-s002.tif]
